# Challenges to communicating the Undetectable equals Untransmittable (U=U) HIV prevention message: Healthcare provider perspectives

**DOI:** 10.1371/journal.pone.0271607

**Published:** 2022-07-21

**Authors:** Daniel Grace, Mackenzie Stewart, Ezra Blaque, Heeho Ryu, Praney Anand, Mark Gaspar, Catherine Worthington, Mark Gilbert

**Affiliations:** 1 Dalla Lana School of Public Health, University of Toronto, Toronto, Canada; 2 Alliance for South Asian AIDS Prevention, Toronto, Canada; 3 School of Public Health and Social Policy, University of Victoria, Victoria, Canada; 4 BC Centre for Disease Control, Vancouver, Canada; University of North Carolina at Chapel Hill, UNITED STATES

## Abstract

“Undetectable equals Untransmittable”, or U=U, is a public health message designed to reduce HIV stigma and help communicate the scientific consensus that HIV cannot be sexually transmitted when a person living with HIV has an undetectable viral load. Between October 2020-February 2021 we conducted 11 in-depth interviews and 3 focus groups with diverse HIV/STI service providers (nurses, public health workers, physicians, frontline providers, and sexual health educators) in Ontario, Canada (n = 18). Our objective was to understand how U=U was communicated to sexual health service users in healthcare interactions. Interview questions were embedded in a larger study focused on improving access to HIV/STI testing. Transcripts were transcribed verbatim and analysed following grounded theory. Most providers emphasized the significance of U=U as a biomedical advancement in HIV prevention but had some challenges communicating U=U in everyday practice. We discovered four interrelated barriers when communicating the U=U message: (1) *provider-perceived challenges with “zero risk” messaging* (e.g., wanting to “leave a margin” of HIV risk); (2) *service users not interested in receiving sexual health information* (e.g., in order to provide “client centered care” some providers do not share U=U messages if service users are only interested in HIV/STI testing or if other discussions must be prioritized); (3) *skepticism and HIV stigma from service users* (e.g., providers explained how the hesitancy of some service users accepting the U=U message was shaped by a legacy of HIV prevention messages and persistent HIV stigma); and (4) *need for more culturally appropriate resources* (e.g., communities other than sexual and gender minority men; non-English speaking service users; that account for broader legal context). We discuss ways to overcome barriers to communicating the U=U message as well as the limitations and potential unintended consequences of U=U framings in the context of unequal access to HIV prevention and treatment.

## Background

Since 2015, many HIV prevention and stigma reduction campaigns have been disseminated internationally that communicate the message that ‘Undetectable equals Untransmittable’ or ‘U=U’ [1, 2]. The scientific grounding of the U=U concept is based on a series of large, methodologically rigorous clinical trials conducted over the last decade [3–6]. Over the past seven years, the HIV stigma reducing as well as scientific implications of HIV undetectability have been incorporated into public health and community messaging through the concise discursive framing of U=U [7–10]. U=U is part of a broader ‘treatment as prevention’ (TasP) strategy that includes pre-exposure prophylaxis (PrEP) and post-exposure prophylaxis (PEP) as other highly effective biomedical tools to prevent HIV transmission using antiretrovirals [11].

Multiple Canadian studies have demonstrated that gay, bisexual, and other men who have sex with men (GBM) have become increasingly exposed to U=U messaging [12–14]. Surveys of Canadian GBM indicate that experiencing social marginalization has a significant negative impact on the diffusion and uptake of the U=U message [14]. Many GBM have experienced sustained HIV-related anxiety as a result of navigating a highly stigmatized epidemic for decades. As such, it is perhaps not surprising that some GBM were not immediately responsive to the prevention message regarding undetectable HIV viral loads despite community discourses encouraging them to do so [15]. Even after the launch of U=U campaigns, qualitative research has observed significant “untransmittable skepticism” by some GBM who are resistant to the U=U framing, and who describe hesitancy and fear associated with having condomless sex with someone living with HIV even if they have an undetectable viral load [12].

Beyond GBM communities, research has indicated that heterosexual adults, including those who may have higher risk for HIV transmission such as Black and Latinx people, have low awareness of the U=U messaging or low confidence in U=U [16, 17]. For example, only 35% of a sample of heterosexuals at high-risk for HIV in New York City were aware of U=U [16], and in Brazil only 17% of respondents believed that U=U was completely accurate [17].

Researchers have argued that the U=U message should be routinely communicated to all patients living with HIV [18]. Calls have also been made for universal patient-provider education regarding U=U regardless of serostatus [7, 8, 19]. However, a number of recent studies have pointed to challenges when communicating this message, including a lack of provider training and education on the concept as well as limited staff resources in some contexts [20]. In other studies, providers have noted that they did not want patients to see U=U as a way to replace other prevention methods such as condoms that could also aid in sexually transmitted infection prevention as well as birth control [21]. Some providers have also described their concern of being responsible if HIV transmission were to happen after providing U=U education to their patients [22].

Given these issues and an uneven adoption of U=U internationally, our objective was to better understand how various HIV/STI service providers (e.g., nurses, public health workers, physicians, frontline providers, and sexual health educators) communicate the U=U message to sexual health service users in Ontario, Canada. We were specifically interested in understanding the communication of the U=U message in everyday practice including the barriers experienced by service providers to consistently convey this HIV prevention message.

## Methods

We used a community-based participatory research (CBPR) framework to guide our study activities [23, 24]. As part of our commitment to CBPR, we hired four peer researchers who self-identified as part of the GBM community and were engaged in all phases of the qualitative study design, including leading interviews and helping to code, interpret, and synthesize study findings. The qualitative research with service providers that we present in this paper occurred in the context of a larger mixed methods study investigating the use and adaptation of online HIV/STI testing services in British Columbia and Ontario, Canada [25, 26]. During in-depth individual interviews and focus groups, service provider participants were asked about the specific benefits and drawbacks an online testing platform may present for GBM, as well as biomedical advancements in HIV prevention. A Community Advisory Board (CAB) comprised of GBM and HIV/STI testing professionals was consulted throughout the process to review the preliminary analysis and help direct the course of research.

Between October 2020 and February 2021, peer researchers conducted 60–90-minute semi-structured individual interviews and focus groups with 18 service providers of HIV/STI testing and other sexual health services such as sexual health nurses, public health workers, physicians, frontline workers, and sexual health educators in Ontario, Canada. We conducted a total of 11 individual interviews and 3 small focus groups (with 2–3 participants per group). The benefit of this focus group format is that it provided participants an opportunity to reflect and engage with the opinions and experiences of their peers. While the study was original designed to be primarily focus groups, many participants were healthcare workers who were on the front lines of the COVID-19 response and took part in individual interviews because of scheduling/availability conflicts. Drawing on the principles of theoretical saturation [27], data collection was completed when no new themes or insights about key study themes emerged in the context of the focus groups and interviews. Due to the social distancing and lockdown measures in place during the COVID-19 pandemic, data collection took place online using Microsoft Teams. We received ethics approval from the University of Toronto Research Ethics Board.

Participants were recruited through purposive sampling [28]. Potential participants were invited to complete an eligibility screener on SurveyMonkey and qualifying participants were selected to participate. Posters advertising the study were distributed through email to AIDS Service Organizations (ASOs), Community Health Centres (CHC), Public Health units, and sexual health clinics that serve sexual and gender minorities within the province. Recruitment posters were also posted online on the social media platform Facebook, specifically within the group Toronto 2SLGBTQ+ Service Providers Network. To be included, participants needed to self-identify as sexual health service professionals in STI/HIV testing and care in Ontario. Participants were excluded if they did not have direct experience working with service users of STI/HIV testing and/or if they currently worked outside of Ontario.

Participants provided their written and informed consent and completed a sociodemographic survey on Qualtrics. During the interview, participants were asked questions regarding their sexual health work experiences, thoughts on online-based testing models and the impact of COVID-19 on sexual health testing services within their organization. We also discussed thoughts on the privacy and personal information of service users, the availability of self and home-testing within the province, their partnerships with private labs, and other questions regarding the promotion of and resources for online HIV/STI testing services.

Participants were asked questions about sexual health communication including U=U messaging. Specifically, we asked: *Do you talk to your clients about U=U and if so*, *how do you communicate undetectability to them*? *What resources do you use to have this conversation*? *What are some of the challenges you experience in having these conversations*? *How do you go about addressing these challenges*? *Do you feel you are equipped to discuss/informed about U=U*? *What kind of resources would you need to have comprehensive conversations about undetectability with your clients*? Within the focus groups and interviews, the terms “client” and “service user” were used synonymously. See S1 File for the full interview guide.

The individual interviews and focus groups were audio recorded and transcribed verbatim. Focus groups were conducted with two peer researchers present to moderate the conversation and take notes. Peer researchers also coded the transcripts using NVivo 12 software following grounded theory techniques [27], developing initial codes and later overarching themes from the data with the consultation of the research coordinator and principal investigator.

## Results

Our 18 participants had a wide range of professional experience including: frontline workers at community-based or HIV/AIDS service organizations (n = 7), sexual health nurses (n = 4), physicians (n = 2), public health workers (n = 2), managers of sexual health nurses (n = 1), clinic counsellors/HIV testing professionals (n = 2). Providers practiced in the Greater Toronto Area (n = 7), Ottawa (n = 2), other large urban areas (> 100,000) (n = 8), and rural communities (1,000–29,000) (n = 1) in Ontario, Canada. Half of participants had over five years of experience in their role (n = 9), followed by 1–5 years of experience (n = 7), and only a few providers had less than one year of experience (n = 2). Participants were asked which populations they primarily served, with the ability to choose more than one option. Providers noted that their service users were primarily GBM (n = 15), people who use intravenous drugs (n = 8) trans/gender non-conforming (n = 7), sex workers (n = 5), youth (n = 2), adults between 18–60 (n = 1), and immigrants/newcomers (n = 1).

The majority of service providers explained that U=U messaging was an important and useful way to help communicate the science of HIV prevention and to counter HIV-related stigma. Many participants described how the U=U mantra was part of the regular “script” that they gave to people testing in sexual health clinics. These providers described having a (loose) script that they used with service users in which U=U messages were integrated. For example, one sexual health nurse explained that while HIV prevention knowledge has improved among some service users, many were still not aware of the U=U message:

…whenever somebody comes for testing, we’ve got our whole spiel, our education spiel. I mean it depends if they haven’t been there before or if they come in regularly then you shorten things up. […] And I talk about U=U, and I’m like oh have you heard about it? I mean it’s getting a little bit better now but there’s still lots that, um, that, don’t know about it. And then, ah, and then oh we’ll go, and they’ll like act surprised or like ‘that’s awesome’, and I—you know just to normalize [HIV] […] you know kind of make it just like normal instead of, um, you know what I mean? It’s not a scary thing anymore, right?

(Focus Group)

This provider then went on to explain how they discussed HIV as a “chronic health condition” like diabetes thanks to the effectiveness of HIV treatment.

Providers also noted how they drew on scientific research findings to help communicate the significance of U=U. Another sexual health nurse put it like this:

I don’t think I have challenges describing it [U=U]. Like I’ve kind of done it enough that I have a bit of spiel, like, the phrases that I use, and I talk about the research, and how there were 22,000, you know, sex acts and no transmission, that kind of puts it in perspective.

(Individual Interview)

This nurse explained that the science behind the U=U message was new to many of the service users they interacted with.

Some providers explained that their communication of U=U was embedded in their routine or standardized assessment process with service users. One public health worker explained it like this:

So, [U=U] comes up in a few different ways. So, for example, in someone’s sexual health assessment we might ask them like, ‘have you been in contact with anyone who has tested positive?’, and they’re like, ‘oh yeah, like my partner has HIV, but he’s undetectable, so no worries’, and we’re like, oh, ‘OK, great’, you know? So that’s great. We also—we do case management on our teams, so for anyone who tests positive for a reportable infection we follow up and it [viral load] also comes up […] ‘this person didn’t tell me that they had HIV, and we had unprotected—or condomless sex,’ and if we look into that and see that they are undetectable, then like there’s no—not much follow-up that we do. And also, if they’re not undetectable, then like our main goal is to try to facilitate getting them onto treatment so that they become undetectable’.

(Individual Interview)

This provider was discussing the work of supporting patients who have sex with someone of a different serostatus and how U=U is discussed in relation to contact tracing. Some providers also emphasised how U=U was “part of the menu of options” and discussed it in relation to other forms of biomedical HIV prevention, noting that pre-exposure prophylaxis (PrEP), post-exposure prophylaxis (PEP), and condoms were also part of/still on the “menu” of HIV prevention. A few providers noted varying degrees of detail they gave regarding U=U messaging, such as providing briefer descriptions of U=U to HIV negative service users or in rooms of mixed serology (e.g., high-level descriptions of treatment efficacy) and more personalized and in-depth U=U counseling with service users living with HIV (e.g., discussion of science behind U=U message; how viral loads work and are monitored).

While a few sexual health nurses said they did not experience major difficulties communicating information on undetectability, most providers encountered one or more challenges when communicating the U=U message. Below we provide a review of four interrelated challenges: (1) *provider-perceived challenges with “zero risk” messaging*; (2) *service users not interested in receiving any sexual health information*; (3) *encountering U=U skepticism and HIV stigma from service users*; and (4) *the need for more culturally appropriate resources accounting for cultural*, *linguistic*, *and medico-legal contexts*.

### (1) Provider-perceived challenges with “zero risk” messaging: Being cautious and leaving a “margin”

A few of the participants we interviewed emphasized their perceived “limitations” to the U=U message, noting that HIV treatment and an undetectable viral load does not result in HIV untransmittability “100% of the time”. One public health worker with extensive clinical nursing team experience described resistance and caution with communicating the message that “zero risk” exists, despite their knowledge that undetectability meant “no realistic risk of transmission”:

We’re kind of in that like space in public health where we’re like a little bit cautious to be like, then there’s ‘zero risk’, because we never talk about anything as ‘zero risk’, even though we can say there’s no realistic risk of transmission if you’re undetectable. So more so it’s just us saying like it’s a great way to greatly, greatly reduce your risk.

(Individual Interview)

This participant explained that they sometimes discuss U=U when service users come in with complaints that their sexual partners did not disclose their HIV status. We return to this theme in the final section of the results, highlighting medico-legal resources that outline when HIV disclosure is not legally required in the context of HIV undetectability. This provider explained it is challenging to talk about U=U because some people living with HIV do not know their viral load status or may be inconsistently on treatment:

Because, you know, really undetectable means you are consistently getting care, and you’re on treatment, and you regularly get your viral load tested so that you know you’re consistently undetectable. So, we just have to be careful with the messaging with that population.

(Individual Interview)

A front-line worker explained needing to “leave a margin” for U=U not being effective. They drew on experience volunteering at an ASO and providing support for people living with HIV:

Well because it’s [U=U] not 100%, it’s 99 whatever, so whenever you leave a little margin of error people will jump on that margin of error, they say, ‘Oh well there’s still a chance. Oh well, you know, it’s not guaranteed’ and, you know, I can’t argue that fact. I can just say well my doctor said I could have sex—I mean I don’t know what it was—like 3,000, sometimes and then one out of those that person would get it, right? So, yeah, I do the best I can, and I can’t change people’s minds for them. All I can do is give them the information, what they do with it, you know, I can’t control that.

(Individual Interview)

To be clear, this understanding of HIV science—the “negligible” or “99 percent” effective frame—is fundamentally at odds with the current scientific consensus regarding HIV being sexual untransmittable when someone has a sustained undetectable viral load [5, 6]. However, this service provider went on to explain how they frequently draw on embodied experience as someone living with HIV when talking about U=U and the importance of HIV treatment: “[I am] proof that it [U=U] works.”

### (2) Service users not interested in receiving sexual health information or information on HIV undetectability

Many of the participants we interviewed emphasised the importance of delivering sexual health information based on the needs and desires of service users. This was frequently framed as “client-centered care” and led some providers to deprioritize conversations about U=U. In some cases, providers said they delayed conversations about U=U after a diagnosis with HIV in order to avoid “information overload” for people, and contextualize this information as part of a larger subsequent discussion. A sexual health clinic manager explained: “so it might be that we talk a little bit about it [U=U] at [the time of HIV diagnosis], but then the next appointment we may do more contact-tracing and have more of a larger discussion. It is a fair amount of information to receive…”. (Individual Interview)

However, some providers said that they delayed discussions about U=U with service users, relaying the message only in very specific circumstances. Some discussed how they did not share U=U information when service users only wanted to get tested for HIV and did not want to receive any further resources. One healthcare provider explained this as such:

Sometimes clients already know all that information, and it’s not really respectful to them or their time to have to sit through it. And sometimes, even if they don’t know the information, sometimes they’re just not receptive, they just, like ‘I’m here because my girlfriend tested positive, she’s forcing me to come in, I don’t want to be here, let’s just get this over with’. […] …they’re not necessarily taking any of it in, and I think that we really need to focus on like that client-centred care of, you know, giving them the information that they want, and asking them if they have any questions, and asking them if they already know about certain topics, and figuring out how much information they want, and really basing it on the client’s needs and wants, rather than just our standard approach.

(Individual Interview)

Another provider drew on their experience as a clinic-based sexual health information counsellor and explained:

If people have more questions about HIV, we would talk more specifically about U=U. It’s not our baseline answer. I mean, a lot of people show up not expecting to have an educational conversation. The majority of our educational talks are to people who are waiting in drop-in for their STI testing appointment. […] Sometimes people are very goal-oriented about STI testing. They’re here to get the thing done and they did not have on their list of things to do also learn about like conversations about partners about transmission. And so, we’re not going to force anybody to do that but also there is some people who end up feeling like it a good opportunity by the end of the session who didn’t necessarily know what that was going to be like going in.

(Individual Interview)

### (3) Encountering U=U skepticism and HIV stigma from service users

Some service providers discussed the mistrust or skepticism with U=U they encountered among some service users—including but not limited to GBM service users—and reflected on how persistent HIV stigma may shape service users’ understanding and acceptance of this HIV prevention message. For example, one HIV/STI testing professional explained how “HIV stigma and all the fear that people have around HIV” resulted in service users being fearful of HIV transmission occurring in sexual encounters without the significant possibility of HIV transmission (e.g., oral sex). This clinic-based social worker went on to explain:

…people don’t know about U=U or kind of this idea that you could be…that effective treatment for HIV eliminates risk of transmission. I feel like maybe like queers know it, or like trans people know that type of thing, or people who are like very interested in this like, in, like sexual health as a field in general, but not so much like the general population. So, people are often really surprised when I mention that [U=U], and I always do at every appointment.

(Individual Interview)

A volunteer at an AIDS Service Organization (ASO) explained U=U “hesitancy” and drew upon CATIE (a national HIV knowledge brokerage organization) messaging in his framing of the “third U” [8]—*universal*:

I think even some people would be hesitant to believe that [U=U is] true because of the stigma attached to it and everything that goes along with it [i.e., HIV and transmission] and I think it’s like racism in the United States, it’s going to take a long time before people finally get it, that yes, you know, U=U. Actually U=U = U because there’s a universal aspect to it.

(Individual Interview)

A front-line service provider working with ethno-racially diverse service users reflected on HIV stigma beyond the Canadian context. They explained that some people living with HIV were also not knowledgeable of the U=U message:

A lot of our clients are either new to Canada and didn’t know the notion of U=U just because of the high stigma and discrimination that happens in other countries, when they come here, they’re really not aware. So that’s one of my jobs […] ‘cause sometimes just for me saying U=U means nothing, ‘cause they’ll still have that stigma that if they sleep with somebody they can still transmit HIV.

(Individual Interview)

### (4) Need for culturally appropriate resources: Communities, languages, and medico-legal contexts

The service providers we interviewed also mentioned a diverse range of U=U resources that they offered to service users, including resources they made in-house (e.g., posters, pamphlets). The organizations most frequently discussed were CATIE (for general/non-population specific U=U resources; https://www.catie.ca), The Prevention Clinic (formerly known as the PrEP Clinic; https://www.preventionclinic.ca/hiv-treatment/u-equals-u/), and The Gay Men’s Sexual Health Alliance (GMSH)—for tailored sexual health messaging for sexual and gender minority men. More specifically, the GMSH’s *TheSexYouWant* campaign was the most commonly named resource; it was described as an incredibly valuable, sex positive, and dynamic source of accessible messaging on U=U and the meaning of HIV undetectability (https://thesexyouwant.ca/). As one public health worker said of the GMSH campaign: “it speaks in such a great tone and it’s really well-received” (Focus Group). Service providers also discussed accessing resources from their local ASOs in Ontario to relay to service users, and referenced the value of being connected with specific HIV care physicians that were “go to” brokers of HIV science information.

A few participants noted needing more accessible, sex positive resources like *TheSexYouWant* for other communities beyond GBM. For example, a clinic educator explained: “But it [*TheSexYouWant*] is specifically aimed at guys who have sex with guys…So if that does not match the person that I am talking to then it’s not as useful” (Individual Interview). The issue of having U=U messages available in languages other than English and French also emerged as an area where more resources are required, as one frontline worker explained: “…like a high number of our clients are all Spanish speaking or Latin American…we refer them to an ethno-specific AIDS service organization, so that they can get the education that they need” (Individual Interview).

Finally, some service providers discussed communicating U=U in the context of the criminalization of HIV non-disclosure. These participants reflected on their work educating service users who were living with HIV about U=U as well as HIV criminalization, drawing on existing medico-legal educational resources (https://www.hivlegalnetwork.ca/; https://www.halco.org/; https://www.catie.ca/uu-a-guide-for-service-providers/hiv-criminalization). Service providers noted that additional culturally appropriate materials may be required to clarify legal responsibilities for HIV disclosure when someone is living with HIV even if they have an undetectable viral load. A few of our participants said they were uncertain about the current legal obligations to disclose one’s HIV status if someone had an undetectable viral load, and discussed the tension between what they knew *scientifically* and what they knew *legally* when talking about U=U with service users. One public health physician put it like this:

…unfortunately I have that dual role of someone in public health knowing all of the kinds of legal issues surrounding, or like the legal background, uh, for HIV, and then I have my own personal clinician views on things and those don’t 100 percent coincide all the time. […] my understanding is people still need to disclose that they’re HIV positive, even though they’re undetectable…if that’s not the case, then I think that’s a gap, and—and would be helpful in terms of just clarifying what the legal responsibilities are for folks. […] Yeah. From the clinician point of view, it’s like U=U, you’re undetectable, don’t worry about the disclosure. Like you—you don’t need to disclose to people if you’re undetectable, type of deal. You’re definitely not infectious, so—but then the whole legal aspect of it is like technically if—like if the law still holds…this is one of the gaps, I’m not too sure where it falls now, that if you are—if you are HIV positive, technically you are supposed to, uh, disclose to your partners. I still recommend disclosing…

(Individual Interview).

## Discussion

The U=U campaign, and the broader scientific message behind the slogan, have had significant uptake both nationally and globally. According to the Prevention Access campaign, “U=U is a thriving and growing community-led movement of HIV advocates, activists, researchers, and over 1,050 Community Partners from 105 countries. Together, we are changing what it means to live and love with HIV around the world” (https://www.preventionaccess.org/). However, we discovered four key challenges service providers encountered that prevented them from consistently communicating the U=U message.

First, some providers described their perceptions of the limitations of the U=U framings and that they wanted to “leave a margin” of risk and were not comfortable with “zero HIV risk” messages. Our findings echo recent research that describes the inconsistency or lack of clarity with which some providers discuss U=U and HIV transmission risk, including those who avoid “no” or “zero” risk language in favour of “negligible” or “(extremely) low” framings [19, 22]. Previous studies have shown that most care providers recommend condom usage for people living with HIV, even if it may undercut U=U messaging [21, 29, 30]; however, it is not always clear from the published literature if providers are making these recommendations in relation to HIV prevention specifically, or STI prevention more generally. Given this inconsistency of messaging from service providers, it is perhaps not surprising that some sexual health service users in the Canadian context adopt similar “negligible” risk framings and forms of “untransmittable skepticism” of the perceived *too-good-to-be-trueness* of the message [12].

Second, participants described that some service users were not interested in receiving sexual health information during their exchanges. In efforts to provide “client centered care”, these service providers said U=U was not always discussed if service users were not interested or had other priorities. Previous studies have reported that some healthcare providers felt as though early TasP discussions could have ethical concerns for patients living with HIV who may not be ready to start medication or be comfortable with the TasP messaging [31]. Our findings reveal a somewhat tailored approach to discussing U=U with some patients and not others. Additional resources may be required to help providers think about when and how to talk about U=U in the context of client-centred care, including when in-depth conversations should be delayed.

Third, some of our participants described the skepticism they encountered regarding the U=U message and HIV stigma from service users. These providers explained how the hesitancy of some service users accepting the U=U message was shaped by a legacy of HIV risk messages and persistent HIV stigma. Tan and colleagues [32] have warned about the unintended harms and stigma that may result from the uncritical celebration and promotion of the U=U message:

…uncritical advocacy of U=U messaging is unwise without close scrutiny from ethics and public health standpoints of how the messaging is promulgated and received. […] If U=U messaging misfires to deepen divides between HIV-negative and HIV-positive individuals or is interpreted as a means of parsing infectious people with HIV from those who have achieved undetectability and uninfectiousness, then U=U messaging will likely have undermined hard-won advances in HIV care, undermined solidarity by designating normal and deviant ways of being a person with HIV, and undermined unity to confer privilege to some and disadvantage to others [32, p.419].

Tan’s concerns of U=U framings echo warnings of the creation of an “undetectable divide” between groups and a stigmatized “viral underclass” [33–36].

As noted in our results, one participant specifically talked about “U=U = U”—drawing upon messaging in the Canadian HIV sector which has highlighted the need for universal access to HIV prevention, and the limits of the HIV prevention message, noting that *viral load does not equal value (V≠V)*:

There are legitimate concerns that the U=U message places inordinate focus on the issue of undetectability and does not address the fact that some people in Canada living with HIV do not have equitable access to ART and to quality, rights-based healthcare. Our collective celebration of U=U is undermined if access to testing, treatment, care, and support—and viral suppression—is not universal [8].

Fourth, participants described the need for more culturally appropriate resources, including materials for populations other than GBM, and for non-English speaking service users. Increased, tailored education on U=U is needed in and beyond the clinical encounter. Many resources have been created to help adapt or frame the U=U message for diverse populations. For example, in the United States a customizable social marketing campaign (with videos, posters, and GIFs) was launched in 2018, allowing users to communicate U=U messages (e.g., can choose one of 9 options) while tailoring languages (5 options) and images (4 options) to meet the needs of the populations they work with (https://positiveseries.org/). U=U has *gone viral*, and the Prevention Access campaign highlights extensive uses and adaptations of the message in different cultural and linguistic contexts globally (https://preventionaccess.org/resources/). Finally, the accounts of our participants revealed tensions with regards to communicating the science of U=U in the context of HIV criminalization in Canada [35, 36].

### Limitations

Many of our participants identified as sexual minorities and experts in HIV prevention which may have influenced their knowledge and perception of U=U in comparison to cisgender, heterosexual providers who neither work with GBM nor specialize in HIV prevention. As such, further research is required to understand the challenges other healthcare providers face, which may be even greater than our participants given the U=U messaging that has been largely targeted towards the GBM community in Canada. If we had interviewed healthcare providers less connected with HIV and sexual health care services, we may have received different accounts, and potentially more skepticism or confusion toward U=U messaging. We did not specifically ask providers how they leveraged both U=U messaging and PrEP simultaneously. More research would be needed to probe further how U=U can have differential impacts on various communities highly affected by HIV and how providers navigate different degrees of knowledge and U=U acceptance—and PrEP use—within and across communities.

## Conclusion

Despite the noted communication challenges, our analysis indicated that U=U is an important tool that many health service providers are utilizing in their clinical encounters. In most cases, it does not appear that providers were sceptical or reluctant to communicate the benefits of undetectable viral load, but there were moments where they had to balance the nuance of the message and the need to reduce HIV stigma with the limited capacity of service users to absorb health information in specific, often highly emotional (potentially stressful) contexts [37]. This was a juggling act. While information on undetectability is becoming more accepted by both providers and GBM, more research is needed to examine if there are differences in the frequency and extent of these challenges to communication and acceptance among different service user groups, and how the balancing of these various priorities can create varied messaging or advice from service user to service user, depending on the assumptions that providers are making. Understanding diverse service user perspectives on U=U communication, including the perceived appropriateness of the messaging received from providers, is necessary. Additional research on other pathways of U=U communication—including how knowledge circulates through social and sexual networks in-person and on-line—is also needed.

Perhaps one of the drawbacks of the rise of U=U as a popular and uniform way of understanding treatment as prevention, is that in creating a sense of an absolute consensus/singular discursive message, it can obscure the nuanced ways providers have to respond to each service user’s unique needs and concerns. Thus, in addition to continuing to evaluate health promotion material on undetectability, our data demonstrate the value of hosting regular (informal) workshops and debriefing opportunities with providers from different organizations and clinics across the province or country, to discuss some of the challenges they may be encountering in relaying new HIV information, and to share some of the successful strategies used to communicate U=U—and advances in biomedical HIV prevention more broadly—with different communities.

Despite a reliance on evidence-based messaging being the standard of care, our work demonstrates that, in practice, such evidence must respond to the service user’s needs and providers must make nuanced decisions about how to translate the significance of treatment as prevention. It is important not to let U=U as a popular discursive or rhetorical frame overshadow this context-specific work and the need to support providers who are working on finding the appropriate balance, but may be reluctant to speak up about their challenges for fear of being perceived as unaccepting of U=U and thus (re)producing HIV stigma. Instead, we should acknowledge the complexity of this shift in orthodoxy in HIV prevention and offer opportunities to providers to navigate the health communication complexities and ethics so that they are best equipped to translate evidence-based messaging in the most impactful way. Finally, the remarkable promise of U=U will not be realized unless structural and material changes are made to ensure access to HIV testing, treatment, and care, comprehensive holistic healthcare, and necessary social supports are universally available.

## Supporting information

S1 File Interview guide. (DOCX)
