# Supplementary material for: Challenges to communicating the Undetectable equals Untransmittable (U=U) HIV prevention message: Healthcare provider perspectives
Source: PLoS One. 2022 Jul 21;17(7):e0271607. doi: 10.1371/journal.pone.0271607 (PMC9302742; doi:10.1371/journal.pone.0271607)
Supplement: S1 File — (DOCX) [file pone.0271607.s001.docx]

**GetCheckedOnline (GCO) Implementation Science Research**

***GCO Acceptability Study***

**Focus Group and Interview Guide for Service Providers of Sexual Health and STI Testing Services**

**Section A: Ice breaker**

A1) Could you please describe your occupation as it relates to sexual health care and/or STI testing?

*Probe: What do you do and where do you work?*

*Probe: What made you interested in participating in this study?*

**Section B: Client Testing Experiences (skip if no testing services are offered, eg. ASO workers)**

B1) Do you currently offer STI and/or HIV testing at your workplace? If yes: Could you walk us through the process a client has to follow to book an appointment for STI and/or HIV testing at your workplace?

Offer only STI or HIV testing? Offering both?

Online reservation? Walk-in? Phone call?

Cost associated with testing? Are clients required to present OHIP/other types of health insurance? What about clients/patients who are not covered by such insurances?

Other services offered at the clinic? Are they integrated?

Has a client ever described any barriers to testing at your workplace?

**Note to facilitators:** “If you currently offer HIV testing to clients: we are interested in understanding how healthcare providers talk about undetectability or U=U in relation to HIV”

B2) B2What do you understand by U=U or undetectability for HIV?

B3) Do you talk to your clients about U=U and if so, how do you communicate undetectability to them?

*Probe:* What resources do you use to have this conversation?

*Probe:* What are some of the challenges you experience in having these conversations?

*Probe:* How do you go about addressing these challenges?

*Probe:* Do you feel you are equipped/informed about U=U?

*Probe:* What kind of resources would you need to have comprehensive conversations about undetectability with your clients?

*Probe:* What resources does your organization offer about U=U?

* * * [Show GCO Video] * * *

**Section C: Thoughts on existing GCO model and online testing**

C1) After having viewed the prototype/video/GCO interface, what are your overall thoughts regarding online-based STBBI testing programs?

*Probe: What are some of the benefits you noted with the program?*

*Probe: What are some of the downsides you noted with the program?*

*Probe: What do you think might be missing?*

*Probe: Is there anything that you noted about the design/interface that you would do differently?*

*Probe: What groups could benefit from this model?*

*Probe: Which tests would you recommend for GCO model testing?*

*Probe: Which tests would you anticipate being harder to do using the GCO model?*

*Probe: What age of service user would you recommend that this testing model serve?*

*Probe: How do you think such a service or program would affect your position or day-to-day work?*

*Probe: Do you think this online model can solve any of the challenges you’ve noted previously?*

*Probe: Are there any concerns you have with this program?*

**Note for facilitators**: “Thank you for your input regarding the GCO model. Now, we will move on to overall thoughts regarding online-based STBBI testing programs in general.

C2) What are some reasons that you think GBQ men, in particular, may benefit from online STBBI testing?

C3) Do you think that GCO/online testing should be widely available to everyone, or would it benefit GBQ men in particular?

*Probe:* ***[IF NOT ADDRESSED BEFORE]*** *Are there other populations who you anticipate could benefit from this program?* ***Additional probe: International students?***

*Probe: In your opinion, are there groups of people that are better served by traditional clinic-based testing models?* ***Additional probe: PLHIV? If yes, why do you think so?***

**Section D: Self and Home Testing**

**Note:** “As discussed in previous sections, online testing might include a GCO-type model. At-home testing may include either only self-collection of samples (which also happens at clinics or labs) or collecting samples and getting your results on the spot - something like a pregnancy test or a diabetes test. This is sometimes called self-testing. With that in mind, let me ask you a few questions about home/self-testing.”

L1) How do you think the availability of self or home testing kits would affect your job or workplace?

*Probe: What ways do you think home or self –testing would effect the services being offered by your and your organization?*

*Probe: What are your concerns around home or self-testing?*

*Probe: What are the benefits for your clients in home or self-testing?*

L2) How do you think home or self-testing kits should be available or delivered to clients?

Through the mail, organizations, pharmacies, doctor’s offices, etc.?

L3) Do you think it’s okay to have costs associated with these kits?

*Probe: How much should they cost?*

*Probe: Where should they be available for purchase?*

**Section E: COVID-19**

D1) How have services at your workplace changed during the COVID-19 pandemic?

*Probe: Have you remained open throughout the lockdown?*

*Probe: How have you scheduled appointments during the pandemic? What’s changed for*  *your clients?*

*Probe: Do you/have you been offering telehealth services? What kind (video calls, telephone)?*

*How has telehealth services changed your assessment of STIs?*

*Probe: How has pre- and post-test counselling changed during the pandemic/lockdown?*

D2) How have conversations about having sex changed throughout the pandemic?

*Probe: Are clients less likely to disclose sexual encounters/behaviours?*

D3) Do you note any changes in people’s testing behaviours due to the pandemic?

*Probe: Is there more reluctance and/or stigma for getting tested/having sex?*

D4) Do you currently offer PrEP-related services at your workplace?

If no: Skip to D5

If yes:

*Probe:* What exactly do you offer? Testing/Prescription/Information/All?

*Probe:* Have there been any disruptions to PrEP services during the pandemic?

*Probe:* Was regular testing related to PrEP delayed? Do you have any examples to share?

*Probe:* Did any of your clients change their PrEP routine?

(Have you heard of any instances of stockpiling/hoarding?)

D5) In what ways do you think an online testing model like GCO might address some of the challenges related to testing during the COVID-19 pandemic?

**Section F: Gauging interest in online testing**

F1) Do you think that your clinic/colleagues would support GCO/online testing? Why or Why not?

F2) What do you think your clients/patients will have to say about GCO/online testing?

*Probe: Do you anticipate that some of your clients/patients would benefit from the availability of GCO/online STI testing?*

*Probe: If they wouldn’t use this service, can you talk about why?*

**Section G: Barriers to implementing online testing**

G1) What are some barriers that you anticipate in implementing GCO/online STI testing in your clinic/organization?

*Probe: What are some facilitators that you would anticipate in implementing GCO/online STI testing in your clinic/organization?*

G2) What would GCO/online testing actually look like? How could it be integrated within your current healthcare delivery system? What would it look like operationally**?**

*Probe:* What are your thoughts on an embedded video conferencing/virtual medicine component for online STI testing?

*Probe:* How would it help operationally?

*Probe:* How would it help legally?

**Section H: Connections to Resources/Services for Clients/Patients**

H1) What supports and infrastructure would you foresee clients needing in regards to accessing an online-based service like GCO?

H2) What are your thoughts on integrating GCO with other services like counselling or PrEP referrals? Would that be beneficial?

*Probe:* How might GCO go about integrating these services?

**Section I: Privacy and Personal Information**

I1) Are there any privacy concerns you would foresee in the GCO model?

I2) What do you think is the best method for delivering test results?

*Probe:* Are there privacy concerns that you or your clients may have with different result delivery methods?

I3) What information do you believe is integral to testing and must be shared by clients, if any?

*Probe: Should clients disclose their gender if the testing is behaviour based?*

*Probe: Should they share their health card number?*

**Section J: Labs**

J1) Does your organization currently work with LifeLabs or DynaCare?

*Probe: What does that partnership look like?*

*Probe: What are the benefits and challenges for working with these labs?*

J2) What about smaller, local community labs?

*Probe: What does that partnership look like?*

*Probe: What are the benefits and challenges for working with these labs?*

**Section K: Promotion**

K1) What would be the best way to inform/advertise to GBQ guys about GCO/online testing?

*Probe: Where do you think these advertisements would be most effective?*

*Probe: What kind of materials/medium would be most accessible?*

**Section M: Closing Remarks**

M1) Is there anything else about testing, online sexual health services, or the specific population(s) you serve that you think is important for us to know with regards to STI testing?

M2) Is there anything you anticipated we would ask about with respect to sexual health/HIV and STI testing that we did not discuss today?
